# Effects of Squat Exercise Variations on Lower Limb Biomechanics and Functional Outcomes: Implications for Physiotherapy and Rehabilitation: A Systematic Review

**DOI:** 10.3390/healthcare14213561

**Published:** 2026-10-22

**Authors:** Bijad S. Alqahtani, Husam A. Almalki

**Affiliations:** Department of Health Rehabilitation, College of Applied Medical Sciences at Shaqra, Shaqra University, Shaqra 11961, Saudi Arabia

**Keywords:** squat exercise, physiotherapy, rehabilitation, musculoskeletal disorders, biomechanics, electromyography, functional outcomes

## Abstract

**Objectives**: To consolidate the evidence regarding the effects of squat exercise variations on lower limb muscle activation, joint kinematics, joint kinetics, force-related outcomes, and functional performance, and to examine their implications for physical therapy, physiotherapy, and rehabilitation. **Methods**: The study conformed to the PRISMA 2020 guidelines. Before the data extraction process started, the protocol for this review was registered in the PROSPERO database (CRD420261390860). A search strategy was developed to systematically review six electronic databases: PubMed/MEDLINE, Embase, Scopus, Web of Science Core Collection, CINAHL Ul-timate, and SPORTDiscus, from inception to 1 April 2026. All articles published in English reporting results of randomized controlled trials and cross-sectional comparative studies involving squat exercise variations in humans were considered eligible. The extracted data pertained to lower limb muscular activation, joint movement, forces, torques, and functional outcomes. Risk of bias was assessed with RoB 2 and a customized appraisal informed by AXIS and JBI, and certainty of evidence was assessed with GRADE. Given the considerable heterogeneity among squat exercises, testing protocols, measurement techniques, and outcome variables, the evidence was synthesized narratively using a structured direction-of-effect approach. **Results**: Twenty-four studies were included: 23 acute cross-sectional comparative investigations, predominantly laboratory-based studies, and one randomized controlled trial; no study recruited a clinical population. The study sample primarily comprised healthy male adults; however, older adults and females were underrepresented. In relation to the synthesis of qualitative literature, deeper squats were associated with greater joint excursion and knee extensor demands, whereas gluteus maximus activation findings varied. Stance width altered hip and knee demands in a protocol-dependent manner. While front squats showed muscle activity similar to back squats, they had a more upright trunk posture and, in one study, less knee compression. Unstable and unilateral squat exercises increased activation of hip stabilization muscles but were accompanied by lower maximal force output. The certainty of evidence was very low for all outcome domains. **Conclusions:** Very low-certainty evidence suggests that squat exercise variations are associated with differences in muscular demands, joint motion, joint loading, and functional performance. Depth, stance, bar placement, stability, and fatigue appear to alter the mechanical characteristics of the exercise differently. These findings may inform individualized squat selection in physical therapy, physiotherapy, and rehabilitation, but longitudinal trials in clinical populations are needed before specific variations can be recommended.

## 1. Introduction

Squats are one of the most commonly employed multi-joint exercises because they involve coordinated movements at the hip, knee, and ankle joints under closed-chain loading [1]. This high prevalence is based on the similarities between squats and many functional movements, such as sitting, standing, stair climbing, landing, braking, and shifting body weight, which all rely on coordinated muscular actions of the lower extremities [2].

In a clinical setting, squat exercises are commonly prescribed to improve lower extremity strength, movement coordination, load tolerance, and the gradual re-establishment of movement patterns [3]. As a result, the squat has become an object of scientific inquiry, not just as an exercise form but as a manipulable movement pattern that can alter muscle activation patterns, joint ranges of motion, joint moments, internal load, and function [1,4].

From a mechanical perspective, the external moment acting at each joint during a squat is the product of the ground reaction force (GRF) and the perpendicular distance between its line of action and the joint center [1,3]. Because the GRF remains close to the midfoot throughout the movement, forward displacement of the knees lengthens the knee moment arm and increases the knee extensor demand, whereas forward inclination of the trunk moves the hip further behind the GRF and increases the hip extensor demand [3]. Restricting anterior knee displacement reduces knee moments but increases hip moments and forward trunk inclination [5], illustrating how small changes in segment alignment redistribute the load between joints.

Although squatting seems to be an easy movement, it demands a high degree of coordination among various aspects, including segment alignment, trunk angle, pelvic and foot positioning, squat depth, load distribution, velocity, and neuromuscular control [2]. Small technical differences can lead to changes in the recruitment of the quadriceps, hamstrings, gluteal muscles, plantar flexors, and hip stabilizers and, hence, changes in the training stimulus and loading of the joints and surrounding structures [3,4].

Some of the commonly applied squat variations include front vs. back loading, high bar vs. low bar placement, wide vs. narrow stance, heel elevation, unilateral vs. bilateral squat, partial vs. deep depth, the use of an unstable surface, and assisted/resisted squats [6,7,8]. Given the differences in loading and joint motion between these variants, their prescription should go beyond the general idea that different squat forms provide the same training effect.

Stance width also involves trade-offs proximal to the hip. A wider stance places the femurs in greater abduction and external rotation and generally reduces the knee flexion and ankle dorsiflexion needed to reach a given depth, whereas a narrower stance requires greater ankle dorsiflexion and anterior knee translation [1,3]. When ankle dorsiflexion or anterior knee translation is limited, depth is attained through additional hip flexion and forward trunk inclination [5], which increases the flexion moment acting on the lumbar spine and the demand on the lumbar extensors to maintain a neutral lumbopelvic posture [3,9]. Direct measurements of lumbar curvature and pelvic tilt across stance widths remain scarce; therefore, the lumbar consequences of stance modification are largely inferred from these trunk-inclination effects.

Squat depth is a principal determinant of knee joint loading. Tibiofemoral and patellofemoral compressive forces increase progressively as the knee flexes and peak near maximal flexion, whereas anterior shear forces, which are restrained mainly by the anterior cruciate ligament, remain low and are confined to the first 60° of flexion [10]. The patellofemoral joint reaction force and stress also increase with knee flexion and external load [11,12,13] because the compressive force increases faster than the patellofemoral contact area at shallow-to-moderate depths [3]. At deeper flexion angles, thigh–calf contact and the “wrapping effect” of the quadriceps tendon may help distribute load [14], and bilateral squatting imposes a relatively low strain on the anterior cruciate ligament compared with non-weight-bearing knee extension [15]. These depth-dependent loading profiles are central to exercise selection after knee injuries or surgery.

Muscle activation patterns are among the most studied variables, as electromyography can reveal squat form biases toward specific muscle groups [4,16]. Nevertheless, it fails to capture the full complexity of mechanical demand because of possible differences in kinematics and kinetics during muscle activation, as well as the lack of a correlation between muscle recruitment and safe loading [17].

Joint kinematics reflect joint motion and, thus, show some aspects of the demand, whereas joint kinetics and force-based measurements reflect mechanical demand itself [3]. In sports and rehabilitation, they are especially important because the prescription of exercises often rests on assumptions regarding patellofemoral and tibiofemoral loads, hip extensor demand, ankle dorsiflexion, and lumbopelvic stability [10,14,18]. However, such assumptions may sometimes contradict the overall body of literature.

Squat-based exercise is also widely used in populations whose movement mechanics deviate from typical patterns. Individuals with patellofemoral pain frequently display altered hip kinematics, including greater hip internal rotation, together with reduced hip muscle strength during weight-bearing tasks [19,20], and dynamic knee valgus during single-leg tasks has been linked to modifiable factors such as hip muscle function and ankle dorsiflexion range [21]. After anterior cruciate ligament reconstruction, patients commonly unload the reconstructed knee during bilateral squatting, producing interlimb asymmetries in knee extensor moments [22]. Selected squat variations are therefore used clinically in the expectation that altering depth, stance, load position, or base of support can move these patterns closer to typical mechanics while keeping joint loads within tolerable limits [3,18]; however, whether specific variations achieve this has not been established systematically.

From a clinical perspective, understanding the biomechanical effects of different squat variations may assist physical therapy and physiotherapy professionals in selecting and modifying exercises for rehabilitation and for individuals with musculoskeletal disorders, particularly when joint loading, movement control, and functional capacity need to be considered.

Interpreting the current results is complicated by variations in populations, testing conditions, EMG normalization procedures, load intensity, squat depth, and methodology [4,17]. Thus, conclusions drawn solely from muscle activation patterns may not be fully justified if other factors, such as joint motion, joint moment, external load, and functional outcome, are ignored.

Several reviews have addressed aspects of this question. Narrative reviews have described squat kinematics and kinetics and their clinical implications [1,3], knee biomechanics during the dynamic squat [10], the effects of depth and load on the knee and spine [14], and differences between high bar and low bar techniques [6]; others have summarized muscle activation during the loaded squat [4], gluteus maximus activation across exercises [16], and the association between squatting and patellofemoral pain [18]. These reviews were narrative, were restricted to a single squat modification, outcome domain, or clinical question, or did not grade the certainty of the evidence. To our knowledge, no previous systematic review has synthesized electromyographic, kinematic, kinetic, force-related, and functional outcomes across the range of squat modifications while appraising risk of bias and certainty of evidence.

Accordingly, this systematic review aimed to determine, in healthy and clinical populations of any age (Population), how modifications and between-participant differences in squat depth, stance width and foot position, bar or load position, unilateral or split execution, surface stability, heel or ankle constraints, and fatigue (Intervention/exposure)—compared with a conventional or alternative squat condition (Comparator)—affect lower limb muscle activation, joint kinematics, joint kinetics, ground reaction force variables, and functional outcomes (Outcomes), as reported in randomized controlled trials and cross-sectional comparative studies (Study design), and to grade the certainty of this evidence.

## 2. Materials and Methods

### 2.1. Review Design

#### 2.1.1. Protocol Development

This review used the PRISMA 2020 reporting guidelines for systematic reviews and meta-analyses [23]. The protocol for this review was registered in the PROSPERO database prior to the commencement of data extraction (CRD420261390860). The review question was formulated using the Population, Exposure, Comparator, Outcomes, and Study Design approach. The population comprised healthy individuals, athletes, physically active adults, and clinical groups who performed squats in lab-based, training, or rehabilitation settings. Because the biomechanics of clinical populations may differ substantially from those of healthy individuals and from one another, the population type was recorded for every study and considered when interpreting the findings. The exposure comprised squat variations or differences defined by modifications to stance width, foot position, squat depth, body position, load position, single or bilateral movement execution, heel lifting, unstable or assisted/restricted conditions, or speed and force generation. The comparator comprised conventional squat technique, different squat variations, a baseline/reference condition, or comparison groups. The outcomes comprised lower limb muscle activity measures, kinematics, kinetics, joint moments, power generation, ground reaction forces, joint/loading parameters, and functional metrics (strength, balance, jump performance, pain, or other capabilities).

The studies included in the review were randomized controlled trials and cross-sectional designs. In this review, “cross-sectional” refers to acute comparative investigations, predominantly within-subject laboratory experiments in which each participant performed two or more squat conditions (repeated measures or crossover designs), and outcomes were measured at a single time point without longitudinal follow-up. Randomized studies were considered because they enable controlled comparison of squat variations, thereby reducing selection bias. Cross-sectional studies were chosen for their widespread use in squat biomechanics research and their applicability to evaluating instantaneous muscle activity, kinematics, kinetics, and force generation across squat variations. Intervention studies without a squat comparator (including uncontrolled pre–post designs), case reports, and noncomparative observational studies were not eligible for inclusion because they would increase methodological heterogeneity and decrease interpretability.

#### 2.1.2. Eligibility Criteria

Peer-reviewed human studies featuring at least one variation in a squat and quantitative data related to lower limb biomechanics or function were eligible for inclusion. Studies had to define the type(s) of squat variations, participant characteristics, and measurement procedures. Reviews, meta-analyses, conference abstracts lacking an accessible full-text version, editorials, opinion articles, animal, cadaveric, and simulator studies lacking validation, and resistance exercise studies excluding squats were excluded.

### 2.2. Search Strategy and Study Selection

A systematic electronic search was performed across PubMed/MEDLINE, Embase, Scopus, Web of Science Core Collection, CINAHL Ultimate, and SPORTDiscus from database inception to 1 April 2026. The included studies were published between 2001 and 2022. Search terms combined controlled vocabulary and keywords arranged in two concept blocks: (1) squat exercise and its named variations, combined using AND with (2) an outcome block in which terms for electromyography, muscle activation, lower-extremity biomechanics, kinematics, kinetics, force-related variables, and functional performance were combined using OR, allowing retrieval through any single outcome domain. Database-specific indexing and syntax were adapted using Medical Subject Headings in PubMed, Emtree in Embase, and controlled vocabulary where available in CINAHL and SPORTDiscus. Boolean operators, truncation, adjacency operators, field restrictions, and phrase matching were modified according to database requirements. The complete database-specific search strategies are provided in Supplementary Table S1, while the completed PRISMA 2020 checklist is presented in Supplementary Table S2.

Records were collated and duplicate records were removed. Two reviewers (B.S.A. and H.A.A.) independently screened titles and abstracts and then the full texts of potentially eligible reports against the eligibility criteria. Disagreements at either stage were resolved through discussion until consensus was reached; no third reviewer was involved. No automation tools were used for screening or eligibility decisions.

### 2.3. Data Extraction

Data were extracted independently by two reviewers using a pre-constructed and pilot-tested form. Disagreements were resolved through discussion until consensus was reached. Extracted items included first author, year, country, study design, participant characteristics, sample size, sex distribution, training or clinical status, squat variation, comparator, load prescription, stance parameters, depth, tempo, surface or equipment use, measurement instrument, electromyographic normalization, kinematic variables, kinetic variables, force-related variables, functional outcomes, assessment time point, numerical results, and statistical parameters relevant for synthesis. When multiple squat conditions were evaluated, only the data relevant to the review question were extracted. During revision, extracted data and study attributions in Table 1, Table 2 and Table 3 were re-checked against the original publications and corrected where necessary.

### 2.4. Risk of Bias and Certainty Assessment

Risk of bias was assessed according to study design. The randomized trial was appraised using the Cochrane Risk of Bias 2 tool [48]. For cross-sectional comparative studies, relevant AXIS items [49] informed seven review-specific domains adapted from the JBI checklist for analytical cross-sectional studies [50]: D1, inclusion criteria; D2, description of participants and setting; D3, valid and reliable measurement and standardization of the squat condition; D4, standardized outcome measurement, including EMG normalization and biomechanical measurement procedures; D5, identification of confounders; D6, strategies to address confounding, including condition order, familiarization, and rest intervals where applicable; and D7, appropriate statistical analysis. The AXIS-informed domain mapping, laboratory-specific confounding criteria, and overall judgment rule constituted a customized appraisal procedure, rather than a validated application of AXIS, JBI, or RoB 2 to these designs. The domains were used to summarize concerns relevant to acute comparative biomechanics studies. Each domain was rated as low, unclear, or high risk. The review-specific overall rule classified a study as low risk when all domains were low, high risk when any domain was high, and unclear otherwise; this was not the RoB 2 algorithm. Two reviewers assessed studies independently and resolved disagreements through discussion. Certainty was assessed separately for each outcome domain using GRADE [51], considering risk of bias, inconsistency, indirectness, imprecision, and publication bias.

### 2.5. Data Synthesis

Given the substantial heterogeneity in study designs, squat protocols, measurement tools, and outcomes, a structured narrative synthesis was undertaken and reported in accordance with the Synthesis Without Meta-analysis (SWiM) guideline [52]. Studies were grouped by the squat modification examined—squat depth; stance width and foot position; bar position and loading configuration (front, back, high bar, low bar, overhead, free-weight, or Smith machine); unilateral, split, and unstable surface variations; ankle, heel, or support constraints (e.g., forefoot wedge, chair, wall, or Spanish squat); and fatigue—and, within each group, by outcome domain: electromyographic, kinematic, kinetic (including joint moments and modeled forces), ground reaction force, and functional or clinical outcomes. When a study reported several outcomes within one domain, the outcome defined by the authors as primary was prioritized; otherwise, peak or whole-repetition values from the heaviest load condition common to all compared variations were used, and each muscle or joint was treated as a separate outcome rather than averaged. For each comparison, the direction of effect (higher, lower, or no difference relative to the comparator) was recorded on the basis of the authors’ statistical comparison, and effect sizes were reported where available. Findings were described as consistent when all studies addressing the same comparison and outcome showed the same direction, and as inconsistent otherwise. Study-reported mean differences, standardized effect sizes, and *p* values were presented where available; no pooled effect measure was calculated.

The feasibility of a meta-analysis was reassessed during revision against the following minimum criteria: at least three studies comparing the same squat variations, reporting the same outcome with comparable normalization or units, and providing extractable means and standard deviations (or correlation coefficients suitable for Fisher z transformation) for a random-effects model. Only the front versus back squat comparison of electromyographic outcomes approached this threshold (three studies; Table 2); however, these studies differed in load (70% of 1 RM, 1 RM, and 10 RM), in the movement phases analyzed, and in the muscles assessed, and the within-participant correlations needed to compute paired effect sizes were not available. Pooling was therefore judged inappropriate, and no subgroup or sensitivity analyses were performed. No formal investigation of heterogeneity, meta-regression, or quantitative reporting bias assessment was undertaken. Reporting bias concerns were considered qualitatively within GRADE and the limitations; funnel plots and statistical small-study-effect tests were not performed.

## 3. Results

### 3.1. Study Selection

**Figure 1:**
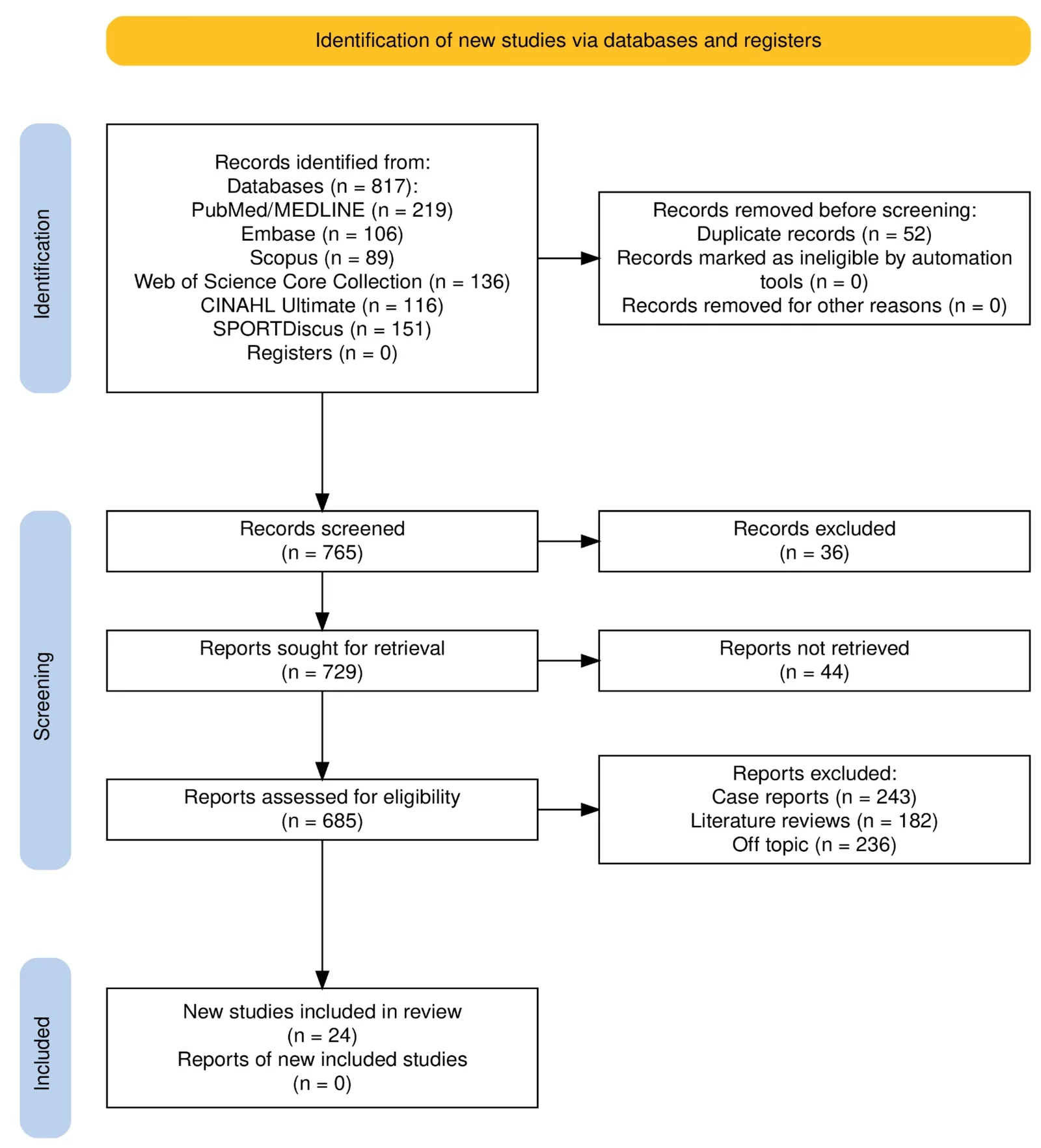
PRISMA flow diagram showing the study selection process.

The database search identified 817 records, and no additional records were found from registers (Figure 1). After 52 duplicates were removed, 765 records were screened by title and abstract. Thirty-six records were excluded at this stage. Of 729 reports sought for retrieval, 44 could not be retrieved because of paywall restrictions. A total of 685 full-text reports were assessed, with exclusions mainly due to case report design (*n* = 243), review-article design (*n* = 182), or off-topic scope (*n* = 236). Twenty-four studies were included in the final review [24,25,26,27,28,29,30,31,32,33,34,35,36,37,38,39,40,41,42,43,44,45,46,47].

### 3.2. Risk of Bias

**Figure 2:**
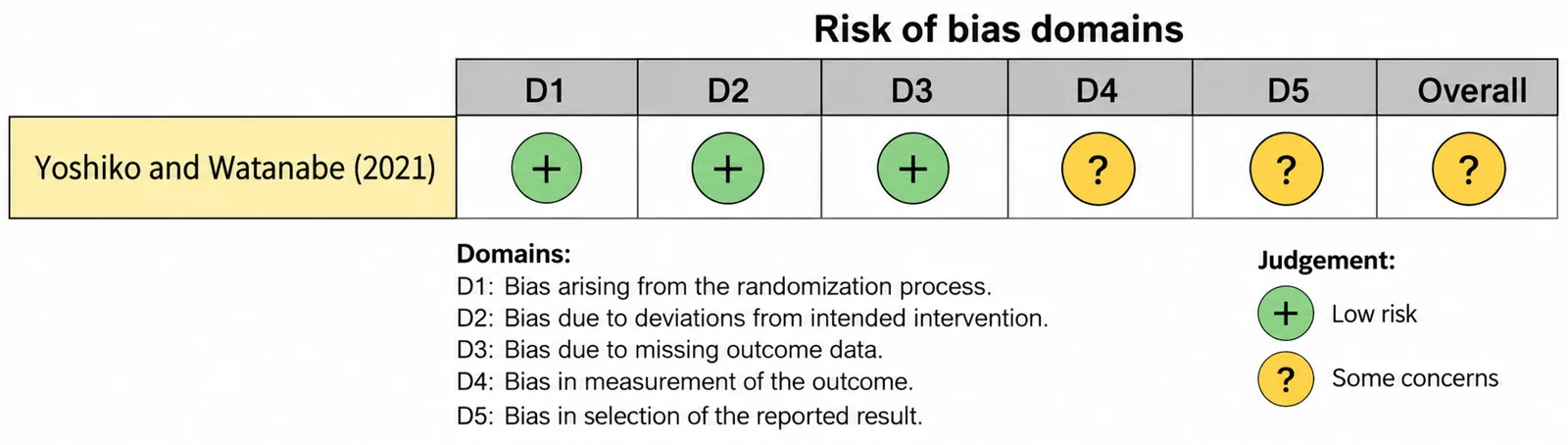
Risk of bias assessment for the randomized comparative training trial [42] using RoB 2.

**Figure 3:**
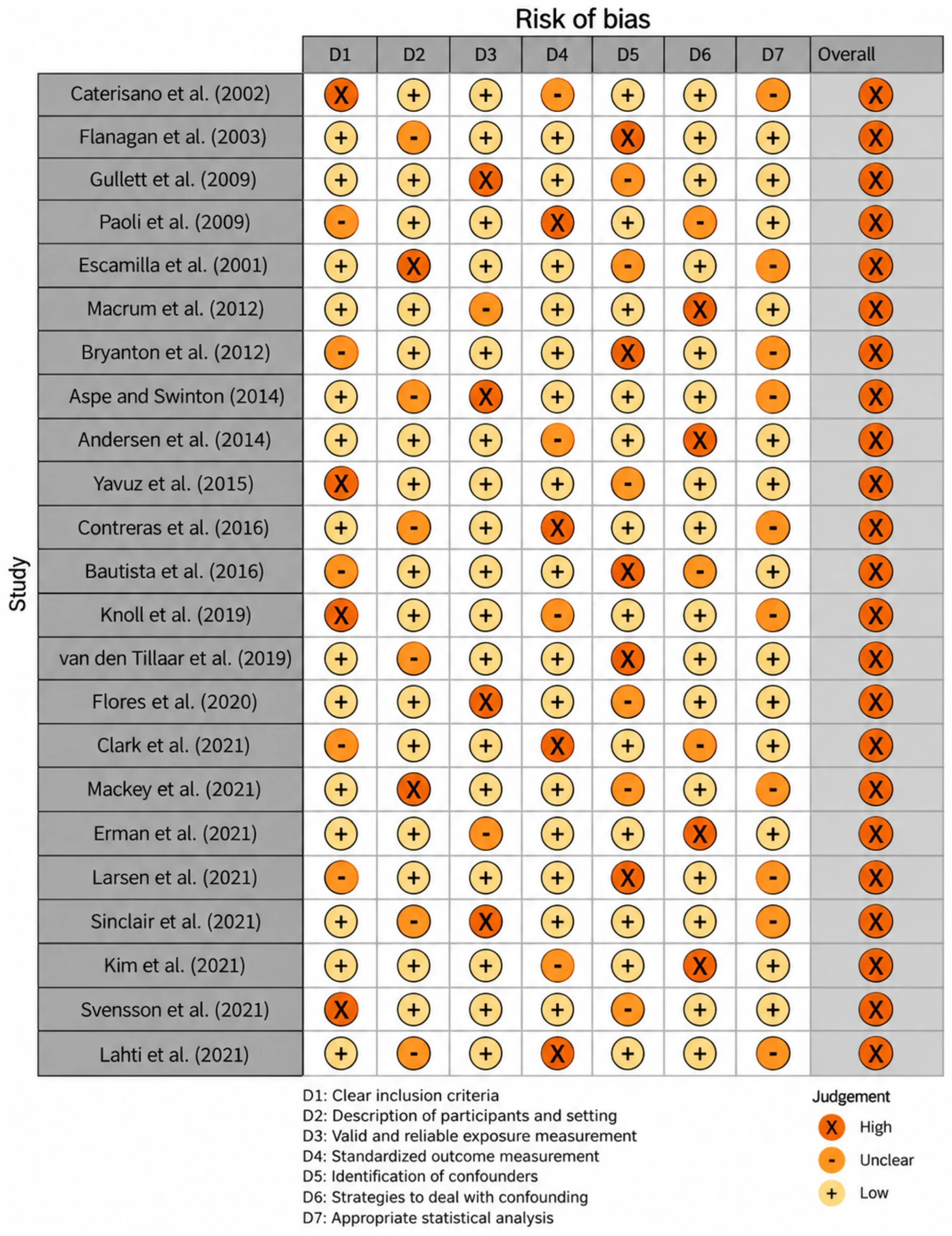
Risk of bias assessment across acute cross-sectional comparative studies using the customized AXIS-informed framework described in Section 2.4. Risk-of-bias assessment of the 23 included studies [29,30,31,32,33,34,35,36,37,38,39,40,41,42,43,44,45,46,48,49,50,51,52]. The assessment was performed across seven domains: D1, clear inclusion criteria; D2, description of participants and setting; D3, valid and reliable exposure measurement; D4, standardized outcome measurement; D5, identification of confounders; D6, strategies to deal with confounding; and D7, appropriate statistical analysis. Risk-of-bias judgments are classified as low (+), unclear (−), or high (×).

The single randomized trial [42] was judged to have some concerns overall, with low risk in randomization, deviations from intended interventions, and missing outcome data, but with some concerns regarding outcome measurement and the selection of the reported result (Figure 2); under the RoB 2 algorithm, some concerns in any domain preclude an overall low-risk judgment. Across the acute cross-sectional comparative studies [24,25,26,27,28,29,30,31,32,33,34,35,36,37,38,39,40,41,43,44,45,46,47], each study had one domain judged at high risk; applying the overall rule described in Section 2.4, all 23 studies were therefore rated at high overall risk of bias (Figure 3). High-risk judgments most often concerned identification of confounders (D5; five studies), followed by inclusion criteria (D1), measurement of the squat condition (D3), standardization of outcome measurement (D4) and strategies to deal with confounding (D6) (four studies each), and description of participants and setting (D2; two studies). No study was at high risk for statistical analysis (D7), although this was the domain most frequently rated unclear (ten studies).

### 3.3. Study and Participant Characteristics

The 24 included reports were published between 2001 and 2022 and were geographically concentrated in the USA, with additional studies from Canada, Norway, Turkey, the UK, Italy, New Zealand, Japan, South Korea, Sweden, and Finland (Table 1). Sample sizes ranged from 6 to 70 participants per study or experiment; Sinclair et al. [44] reported separate experiments with 70 and 20 participants. Most samples comprised healthy adults, resistance-trained individuals, or athletes. Two reports recruited older adults [25,42], while female-only and mixed-sex samples were less common than male-only samples. Study-specific demographic data are presented in Table 1. Although clinical populations were eligible, no included study recruited a clinical population. This limited the applicability of the findings to rehabilitation patients.

### 3.4. Methodological Characteristics

Twenty-three reports described acute comparative investigations, predominantly laboratory studies with within-subject conditions; ref. [28] was a competition-based between-participant comparison, and [44] included both between-participant and repeated measures experiments. One report was a 12-week randomized comparative training trial using stratified semi-random assignment [42] (Table 1 and Table 2). Surface electromyography was the most common method for assessing muscle activation, whereas mechanical outcomes were assessed using force plates, motion-capture systems, inverse dynamics, musculoskeletal modeling, ultrasonography, and dynamometry. Loading ranged from bodyweight conditions to high-intensity protocols, including 3-repetition maximum, 4-repetition maximum, 6-repetition maximum, 10-repetition maximum, 12-repetition maximum, 70–85% of one-repetition maximum, and maximal one-repetition testing. Squat depth varied across partial, parallel, below-parallel, full, self-selected, and externally constrained conditions. Maximal voluntary isometric contraction was the most frequently used electromyographic normalization method, although kinetic and modeling studies did not require it.

The main outcome domains were muscle activation [24,25,26,27,29,31,32,33,34,35,36,37,39,43,45,46], joint kinematics [24,25,28,29,30,33,37,38,40,41,43,44,47], joint kinetics or moments [25,26,28,30,38,40,43,44,47], and ground reaction force variables [31,37,41,43,44]. Functional outcome assessment was uncommon and was mainly represented by fatigue-induced repetition ability, strength/hypertrophy outcomes, or center-of-pressure measures of stability. Overall, the evidence was more focused on acute neuromuscular and biomechanical responses than on long-term clinical or performance adaptations.

### 3.5. Outcome Synthesis

Findings are summarized below by outcome domain; study-level results are presented in Table 3, and each direction of effect is expressed relative to the stated comparator.

#### 3.5.1. Electromyographic Outcomes

Sixteen studies reported muscle activation [24,25,26,27,29,31,32,33,34,35,36,37,39,43,45,46]. For squat depth, relative gluteus maximus EMG contribution increased with depth in one study [24], whereas no differences in gluteus maximus, biceps femoris, or vastus lateralis activity were found among parallel, full, and front squats in trained females [34]. Wider stances increased gluteus maximus [27,43] and adductor activity [43], while modeling identified stance-related differences in quadriceps and posterior chain muscle forces [44]. Front and back squats produced broadly similar lower limb activation [26,34], although the vastus medialis was more active in the front squat and the semitendinosus in the back squat during the ascending phase [33]. Low bar placement increased erector spinae activity and high bar placement increased vastus lateralis activity in the post-sticking region [43]; the back squat elicited greater posterior trunk and lower-body activity than the overhead squat, which elicited greater anterior trunk activity during descent [31], and the overhead squat elicited greater middle trapezius activity than the front squat [35]. Unstable surface and unilateral variations increased gluteus medius [32] or gluteus maximus activation [36], and free-weight unilateral squats elicited higher activity in several muscles than Smith machine variants [37], and free-path bilateral squats elicited greater whole-squat quadriceps activation and phase-specific hamstring activation than fixed-path squats, with no gluteus maximus difference [46]. Restricting ankle dorsiflexion reduced vastus lateralis and vastus medialis activity and increased soleus activity [29]; the chair squat shifted activity toward the biceps femoris and gluteus maximus in older adults [25]; the Spanish squat elicited the highest rectus femoris activity among three bodyweight variations [45]; and vastus lateralis and trunk muscle activity increased during a fatiguing set [39].

#### 3.5.2. Kinematic Outcomes

Thirteen studies reported kinematic outcomes [24,25,28,29,30,33,37,38,40,41,43,44,47]. Greater squat depth was consistently accompanied by greater hip and knee flexion [24,30,38]. Stance width comparisons showed protocol-dependent differences in hip and knee motion [28,44,47]. Lahti et al. reported greater knee flexion with a narrow stance, but no statistically significant hip flexion difference under their corrected threshold [47]. The back squat [33] and low bar placement [43] were associated with greater trunk inclination than the front squat and high bar placement, respectively. Restricting ankle dorsiflexion with a forefoot wedge reduced peak knee flexion and increased knee valgus and medial knee displacement (effect size up to 2.92) [29]. Compared with the conventional squat, the chair squat increased hip flexion and reduced knee and ankle motion in older adults [25], and the Bulgarian split squat increased peak ankle displacement, whereas the back squat increased peak knee displacement [40]. Fatigue increased hip, knee, and ankle range of motion [41], and barbell velocity profiles differed between free-weight and Smith machine unilateral squats [37].

#### 3.5.3. Kinetic and Ground Reaction Force Outcomes

Nine studies reported joint kinetics or modeled loading [25,26,28,30,38,40,43,44,47], and five reported ground reaction force variables [31,37,41,43,44]. The peak external knee flexion moment increased with both depth and load, and load reductions at greater depth did not offset this increase [38]; torque-derived relative hip extensor effort increased with both depth and load, whereas relative knee extensor effort increased with depth but not load [30]. Wider stances were associated with greater hip and knee moments in the competition-based comparison [28], while Lahti et al. reported a greater hip-to-knee extension moment ratio and greater knee adduction moments with a wide stance [47], although a musculoskeletal modeling study found no differences in sagittal joint moments across stance widths [44]. The back squat produced higher knee compressive forces and knee extensor moments than the front squat (1.0 ± 0.4 vs. 0.7 ± 0.2 N·m/kg) [26]. The chair squat increased hip extensor moments, whereas the conventional squat increased knee extensor and ankle plantar flexor moments [25], and the Bulgarian split squat was more hip-dominant, with lower relative knee demand, than the back squat [40]. Peak force was higher in the back squat than in the overhead squat [31], in low bar and wide-stance conditions [43], and in the Smith machine than in free-weight unilateral squats [37]; wider stances increased the medial ground reaction force impulse [44], and fatigue altered the forefoot–rearfoot distribution of ground reaction force [41].

#### 3.5.4. Functional and Clinical Outcomes

Functional, fatigue, or stability-related outcomes were reported in five studies [32,36,39,41,42]. In the only longitudinal trial, Yoshiko and Watanabe compared 12 weeks of shallow and deep home-based squat training in 16 older adults [42]. Neither group showed significant changes in maximal isometric knee extensor torque, quadriceps thickness, or walking speed (*p* > 0.05). Sit-to-stand performance and leg-press 1 RM improved from baseline in both groups at selected time points (*p* < 0.05), without evidence that deep squats were superior overall. The remaining evidence was acute: an unstable surface reduced the 6 RM load [32], center-of-pressure displacement differed between single-leg and split–squat variations [36], repetition performance differed by training status [39], and fatigue altered joint excursion and force distribution [41]. No included study assessed patient-reported function, injury incidence, or return-to-activity outcomes, and none recruited a clinical population.

### 3.6. Certainty of Evidence

According to the GRADE assessment (Table 4 and Table 5), the certainty of the evidence was very low across all major domains, primarily due to the prevalence of short-term laboratory cross-sectional or repeated measures studies, with only one randomized trial identified [42]. The evidence for muscle activation was reduced due to small, heterogeneous EMG studies conducted at various squat depths, stance widths, loads, bar positions, unilateral versus bilateral conditions, and EMG normalization techniques [24,25,26,27,29,31,32,33,34,35,36,37,39,43,45,46]. Although deeper squats increased relative gluteus maximus EMG contribution in one study [24] and torque-derived hip extensor effort in another [30], a wider stance increased hip extensor and adductor muscle contribution [27,43,47], while both unilateral and unstable conditions increased the need for stabilizers [32,36,37], the certainty of these findings remained very low since they were not obtained in well-controlled trials with sufficient generalizability. The joint kinematics findings were also rated very low, as changes in hip, knee, and ankle movement depended on depth, stance width, fatigue status, and external load conditions [24,25,28,29,30,33,37,38,40,41,43,44,47]. Greater squat depth required greater joint excursion [24,30,38], while stance width findings varied by protocol [28,43,44,47]. With respect to joint kinetics and loading, the certainty of findings remained very low because only a portion of studies investigated joint moments, joint compression, kinetics, and modeled force values [25,26,28,30,38,40,43,44,47]. As seen, front squats may lead to lower knee compressive loads than back squats [26], whereas deeper, heavier squats were associated with greater demands on knee extensor moment generation [38]. A wide stance altered hip-to-knee demand, but did not consistently reduce absolute knee loading [28,43,47]. The ground reaction force findings were particularly sparse, with only a handful of studies evaluating force distribution or impulse values [31,37,41,43,44], in which changes depended on fatigue, stance width, bar position, and machine/free-weight conditions. The functional evidence was least convincing and indirect, based on only a few trials investigating muscle activation, fatigue, balance, strength gains, or hypertrophy effects [32,36,39,41,42]. The longitudinal trial found no significant increase in knee extensor torque or muscle thickness and did not establish superiority of deep over shallow squats [42]. Other functional findings relied largely on acute or indirect variables, including 6 RM load, center-of-pressure displacement, and fatigue responses [32,36,39,41].

## 4. Discussion

The findings of this review suggest that different squat variations are associated with a redistribution of neuromuscular and mechanical demands among the lower limb and trunk joints. Factors such as squat depth, stance width, barbell placement, execution of a single-leg version, incorporation of instability elements, and fatigue affect the distribution of joint load and muscle activation rather than the movement itself. Deeper squats involved greater joint excursion; relative gluteus maximus EMG contribution increased in one comparison [24], whereas another reported no depth-related EMG difference [34]. Wider-stance effects on hip and knee demands varied by protocol. These patterns are consistent with earlier biomechanical reviews [1,3,14]. However, they derive almost entirely from acute laboratory comparisons in healthy, mostly trained adults, and the certainty of evidence was very low for every outcome domain; they therefore describe acute mechanical demand rather than demonstrating that any variation improves training or rehabilitation outcomes.

Front squats and back squats elicited similar overall muscle activity in the lower limbs. However, front squats were associated with a more upright trunk and, in the single kinetic comparison available [26], less knee compressive loading compared to back squats. From a clinical perspective, this distinction is relevant because identical muscle activation patterns do not imply identical joint loading. For individuals who need to avoid excessive knee compressive loading and trunk inclination during a squat, front squats may be a reasonable alternative to regular back squats, provided that the movement can be executed properly [3]; however, this inference is based on one study in healthy trained adults and has not been tested in clinical populations.

Both unstable and unilateral squat variations increased stabilizer demand but were accompanied by lower maximal force output. Such trade-offs are consistent with the broader literature on instability resistance training [8,53,54] and may be relevant when designing programs aimed at rehabilitation and performance enhancement. These acute changes suggest hypotheses about stabilizer demand and load capacity; they do not demonstrate improved balance, rehabilitation recovery, or injury prevention. Clinical selection requires patient-specific assessment because no included study tested these outcomes in patients.

Bar placement and stance width also have implications for trunk stability. Low bar placement was associated with greater forward trunk inclination than high bar placement [43], in line with previous analyses [6], and the back squat was associated with greater trunk inclination than front squat [33]. Greater trunk inclination lengthens the horizontal distance between the lumbar spine and the combined center of mass of the upper body and barbell, increasing the lumbar extensor moment and spinal compressive loading [3,9]. With the lumbar segments more inclined relative to the vertical, a larger share of this load acts as anterior shear. Wide-stance, low bar squatting, which is commonly adopted in powerlifting, combines these features [6]. However, the included stance width studies mainly reported hip and knee changes [28,43,44,47], and none directly measured lumbar kinematics or spinal loading. When the bar position and stance width are selected for individuals with low back pain or limited trunk extensor capacity, these potential lumbar demands warrant consideration.

External biomechanical studies have reported that patellofemoral force and stress increase with knee flexion and external load [3,11,12,13], and the included healthy-participant study also found higher external knee flexion moments with greater depth and load [38]. These findings did not establish a contraindication, safe flexion threshold, or injury risk in patients. External clinical guidance supports exercise therapy for patellofemoral pain [55], with squat depth and load adjusted to symptoms and individual tolerance [3]. Such clinical considerations derive from external literature, not from evidence of rehabilitation effectiveness generated by this review. The present synthesis did not support variation-specific clinical contraindications.

However, the generalizability of these findings is limited by the preponderance of young, healthy, resistance-trained male participants. The findings cannot be assumed to apply to females, older adults, novice trainees, injured or diseased individuals, or those with hip, knee, ankle, or neuromuscular conditions, and no included study recruited a clinical population. In particular, patellofemoral pain, hip strength deficits, ankle dorsiflexion limitation, fatigue, and poor force generation might affect squat mechanics and loading distribution patterns [19,20,21,22,29].

Further investigations into the influence of various squat modifications on squat mechanics are needed. Future research should prioritize: employing larger sample sizes and incorporating more clinical populations; establishing universal definitions of squat depth; normalizing muscle activation levels uniformly across studies; integrating the reports on kinematics, kinetics, muscle activation, and functional outcomes in each study; conducting randomized longitudinal trials, including trials in clinical populations with pain, function, and patient-reported outcomes, since the existing literature includes mostly the analyses of acute biomechanical responses to squatting; and finally, evaluating sex-, age-, and symptom-based responses to the squat modifications [3,17].

### 4.1. Limitations

This review has several limitations. Heterogeneity in squat variations, loading regimes, squat depth definitions (defined variously by thigh orientation, knee angle, or chair or target height), testing methods, and normalization procedures (MVIC in most studies, but raw RMS, peak dynamic EMG, relative contribution to summed EMG, or unspecified methods in others) prevented the conduct of a meta-analysis, and the direction-of-effect synthesis used instead conveys neither the magnitude nor the precision of effects and gives equal weight to small and larger studies. In addition, most studies had small sample sizes (6–70 participants), predominantly recruited healthy, resistance-trained adults, and focused on the acute biomechanical response to squat modifications; only one randomized trial provided longitudinal data, and no study recruited a clinical population, so the evidence retrieved was narrower than the population eligibility criteria. Selective outcome reporting within studies could not be assessed against pre-registered protocols and therefore cannot be excluded, and small-study effects could not be assessed formally. Forty-four reports could not be retrieved, and restriction to English-language publications may have excluded eligible studies; both could have introduced selection bias. Finally, AXIS was developed for cross-sectional studies rather than specifically for laboratory comparisons. The customized domain mapping and overall judgment rule (Section 2.4) were not independently validated and required reviewer judgment.

### 4.2. Practical Considerations

Within the limits of very low-certainty evidence, squat variations may be selected according to the intended biomechanical or clinical target. Deeper squats may increase hip extensor and gluteus maximus demand, but they also increase joint excursion and knee extensor demand, which may be undesirable when these need to be limited. Stance width may redistribute hip and knee demands, but wider stances cannot be assumed to reduce knee loading across protocols. Unilateral and unstable squats may be considered when targeting balance and stabilizing muscle activity, whereas stable bilateral squats may be preferred when maximal force production is the objective. Finally, restricted ankle dorsiflexion was associated with greater medial knee displacement [29], and fatigue altered joint excursion and ground reaction force distribution [41]; movement quality may therefore warrant closer monitoring in these situations. These considerations should be regarded as hypotheses for clinical testing rather than as evidence-based prescriptions.

## 5. Conclusions

Based on very low-certainty evidence derived mainly from acute laboratory studies in healthy, predominantly male and trained adults, squat variations were associated with differences in muscle activity, joint movement, joint loading, force distribution, and several functional parameters. No single variation appeared superior across all outcomes; rather, each variation altered the mechanics of the squat differently. Greater squat depth increased joint excursion and knee extensor demand, while EMG responses varied. Wider-stance effects on hip and knee loading depended on the protocol and did not establish a consistent reduction in knee loading. Bar placement and squat type were also associated with differences in the movement strategy adopted during this exercise. In particular, front squats were similar to back squats in muscle activation, but were characterized by a more upright trunk and, in one study, lower knee compressive forces. Unilateral squats, split squats, and unstable surface squats elicited greater stabilizing muscle activity, especially in the hip muscles, but were accompanied by lower maximal force production compared with bilateral squats. Lastly, fatigue was associated with increased muscle activity and joint excursion and with altered ground reaction forces. These findings may inform, but cannot yet determine, squat selection in physiotherapy and rehabilitation; randomized longitudinal trials in clinical populations are needed before variation-specific clinical recommendations can be made.

## Figures and Tables

**Table 1:** Demographic variables of included studies.

Author(s)	Year	Country	Study Design	Sample Size (*n*)	Age (Mean ± SD)	Sex (M/F)	BMI/Body Mass (kg)	Height (cm)	Training Status	Population Type
Caterisano et al. [24]	2002	USA	Cross-sectional	10	23.0 ± 3.0	10 M/0 F	82.7 ± 11.8 kg	178.0 ± 6.5	Resistance-trained (min 5 yrs experience)	Healthy young adults
Flanagan et al. [25]	2003	USA	Cross-sectional (repeated measures)	22	74.5 ± 4.39	9 M/13 F	74.1 ± 12.7 kg	166.0 ± 9.0	Healthy community-dwelling	Older adults (70–85 yrs)
Gullett et al. [26]	2009	USA	Crossover	15	22.1 ± 3.6	9 M/6 F	69.7 ± 6.2 kg	171.2 ± 6.4	At least 1 year of both front and back squats	Healthy trained adults
Paoli et al. [27]	2009	Italy	Cross-sectional	6	26.3 ± 3.6	6 M/0 F	79.5 ± 6.7 kg	177.0 ± 4.0	Experienced lifters (>2 yrs)	Healthy trained males
Escamilla et al. [28]	2001	USA	Cross-sectional (between-participant, competition-based)	39	NE	39 M/0 F	NE	NE	Competitive powerlifters	Competitive powerlifters
Macrum et al. [29]	2012	USA	Cross-sectional	30	22.3 ± 2.8	15 M/15 F	NR	NR	Recreationally active	Healthy young adults
Bryanton et al. [30]	2012	Canada	Cross-sectional (repeated measures)	10	24.5 ± 3.2	0 M/10 F	73.7 ± 12.2 kg	170.0 ± 9.0	Resistance-trained (min 1 yr)	Healthy strength-trained women
Aspe and Swinton [31]	2014	UK	Cross-sectional (within-subjects crossover)	14	26.0 ± 7.0	14 M/0 F	90.5 ± 17.5 kg	182.5 ± 13.5	Experienced with BS & OHS	Healthy trained males
Andersen et al. [32]	2014	Norway	Cross-sectional (repeated measures)	15	25.0 ± 4.0	15 M/0 F	79.7 ± 8.2 kg	179.6 ± 5.8	Resistance-trained (>6 months)	Healthy trained males
Yavuz et al. [33]	2015	Turkey	Cross-sectional	12	21.2 ± 1.9	12 M/0 F	72.5 ± 8.2 kg	174.0 ± 6.0	Experienced with squat exercises	Healthy trained males
Contreras et al. [34]	2016	New Zealand	Cross-sectional	13	28.9 ± 5.1	0 M/13 F	58.2 ± 6.4 kg	164.0 ± 6.3	Resistance-trained (min 1 yr)	Healthy trained females
Bautista et al. [35]	2020	USA	Cross-sectional (within-subjects crossover)	7	28.0 ± 3.6	7 M/0 F	92.0 ± 26.1 kg	175.0 ± 5.3	Recreationally trained (>1 yr, >3 x/wk)	Healthy trained males
Knoll et al. [36]	2019	USA	Cross-sectional (repeated measures)	17	20.5 ± 1.3	0 M/17 F	66.2 ± 10.1 kg	166.0 ± 5.7	Recreationally active females	Healthy young females
van den Tillaar et al. [37]	2020	Norway	Cross-sectional (repeated measures)	14	25.0 ± 4.0	14 M/0 F	80.2 ± 9.3 kg	179.0 ± 6.8	Resistance-trained (>2 yrs)	Healthy trained males
Flores et al. [38]	2020	USA	Cross-sectional (repeated measures)	16	22.1 ± 2.2	0 M/16 F	66.7 ± 8.9 kg	163.5 ± 5.9	Recreationally trained women	Healthy trained females
Clark et al. [39]	2021	UK	Cross-sectional	40	26.0 ± 6.0	40 M/0F	NR	NR	Novice (*n* = 21) vs. Experienced (*n* = 19)	Healthy males (novice + trained)
Mackey et al. [40]	2021	USA	Cross-sectional (repeated measures)	20	24.2 ± 2.5	20 M/0 F	85.3 ± 13.9 kg	176.0 ± 6.0	Resistance-trained	Healthy trained males
Erman et al. [41]	2021	Turkey	Cross-sectional	20	24.0 ± 3.1	20 M/0 F	78.7 ± 11.5 kg	178.9 ± 7.1	Recreational athletes	Healthy male recreational athletes
Yoshiko and Watanabe [42]	2021	Japan	Randomized comparative trial (stratified semi-random assignment)	16	SS: 71.0 ± 4.0; DS: 68.6 ± 3.6	3 M/13 F	22.6 ± 2.9 (BMI)	NR	Community-dwelling; activity levels varied	Older adults (≥65 yrs)
Larsen et al. [43]	2021	Norway	Cross-sectional (repeated measures)	15	27.0 ± 5.0	15 M/0 F	84.5 ± 10.8 kg	180.0 ± 6.2	Resistance-trained (>3 yrs)	Healthy trained males
Sinclair et al. [44]	2022	UK	Two experiments: between-participant and repeated measures	Exp1: 70; Exp2: 20	Exp1: 29.25 ± 5.40; Exp2: 32.10 ± 10.02	70 M/0 F; 20 M/0 F	Exp1: 81.14 ± 9.88 kg; Exp2: 89.95 ± 13.21 kg	Exp1: 177.25 ± 5.76; Exp2: 182.10 ± 4.88	Regular squatters	Healthy trained males
Kim et al. [45]	2022	South Korea	Cross-sectional	22	23.2 ± 2.5	14 M/8 F	NR	NR	Physically active university students	Healthy young adults
Svensson et al. [46]	2022	Sweden	Cross-sectional	16	22.8 ± 4.3	10 M/6 F	67.7 ± 8.9 kg	174 ± 6	Athletes (>2 yrs training)	Healthy athletes
Lahti et al. [47]	2019	Finland	Cross-sectional	14	NE	NE	NE	NE	Amateur rugby athletes	Healthy trained males

Note: Twenty-three reports described acute cross-sectional comparative investigations, predominantly within-subject laboratory comparisons; the competition-based comparison [28] and the between-participant component of [44] were distinguished from repeated measures experiments. One report described a randomized comparative training trial [42]. NR, not reported; NE, not extracted from the verified source; RCT, randomized controlled trial. Appraisal classifications are described in Section 2.4.

**Table 2:** Methodological variables of included studies.

Author(s)	Squat Variation(s) Compared	Loading Protocol	Squat Depth	Equipment Used	EMG System/Measurement Tool	Muscles Assessed	Kinematic/Kinetic Analysis Method	No. of Trials/Repetitions	Normalization Method
Caterisano et al. [24]	Partial, parallel, full back squat	Body weight + barbell (various loads)	Partial (above 90°), parallel (90°), full (below 90°)	Barbell, squat rack	Surface EMG	VL, VM, BF, GM	2D video analysis	3 reps per condition	Relative contribution to summed EMG of the four muscles
Flanagan et al. [25]	Normal squat (SQ) vs. Chair squat (CSQ)	Body weight	SQ: self-selected; CSQ: 43.8 cm chair height	Force plate, chair	Surface EMG + motion capture	VL, VM, BF, ST, MG, SOL, TA, RF	Inverse dynamics (8-camera motion analysis)	3 trials each	MVIC
Gullett et al. [26]	Back squat vs. Front squat	70% of the 1 RM determined separately for each squat variation	To 90° knee flexion	Barbell, force plate, 2 video cameras	Surface EMG (900 Hz)	RF, VL, VM, BF, ST, ES	3D video + force plate; inverse dynamics	2 trials × 3 reps per variation; second repetition analyzed	MVIC
Paoli et al. [27]	Back squat at narrow, medium, wide stance	0%, 30%, 70% 1 RM	Parallel depth (thighs parallel)	Barbell, squat rack	Surface EMG	VM, VL, RF, ST, BF, GM, GMed, Add	Video monitoring for depth	10 reps × 3 sets per condition	Not normalized to MVIC (raw RMS)
Escamilla et al. [28]	Self-selected narrow, medium and wide competition stance groups	National powerlifting championship attempts	Competition squats; analyzed at 45°, 90° and maximal knee flexion	Barbell; two 60 Hz cameras	Video-based biomechanical analysis; no EMG	No EMG muscles assessed	2D and 3D video analysis; calculated net joint moments	NE	Not applicable to EMG
Macrum et al. [29]	Double-leg squat: no-wedge vs. 12° forefoot wedge	Body weight	To max depth achievable	12° forefoot wedge, motion capture, force plate	Surface EMG	VMO, VL, LG, SOL	3D kinematics (motion capture) + force plate	7 trials per condition	MVIC
Bryanton et al. [30]	Back squat at varying depths + loads	50%, 60%, 70%, 80%, 90% 1 RM	Partial, parallel, full	Barbell, motion capture, force platform	Motion analysis and joint-angle-specific maximal isometric torque; no EMG	Hip extensors, knee extensors and ankle plantar flexors (torque-derived effort)	3D inverse dynamics	5 reps per condition	Net joint moment/angle-matched maximum voluntary torque
Aspe and Swinton [31]	Back squat vs. Overhead squat (60%, 75%, 90% 3 RM)	60%, 75%, 90% of 3 RM	Below parallel	Barbell, force platform	Surface EMG	AD, RA, EO, ES, GM, VL, BF, GA	Force platform kinetics	3 RM attempts	MVIC
Andersen et al. [32]	Bilateral squat vs. Bulgarian squat (stable vs. unstable surface)	6 RM load	Parallel depth (~90°)	Barbell, BOSU, force plate	Surface EMG	VL, VM, RF, GM, GMed, BF, ST, Sol, Add	Force plate	6 reps per condition	MVIC
Yavuz et al. [33]	Front squat vs. Back squat	Maximal load (1 RM)	Thighs parallel to ground	Barbell, 2D video system	Surface EMG	VL, VM, RF, ST, BF, GM, ES	2D kinematic analysis	3 max-load attempts	MVIC
Contreras et al. [34]	Parallel squat vs. Full squat vs. Front squat	10 RM load per variation	Parallel (thighs ||), Full (below ||), Front squat (below ||)	Barbell	Surface EMG	Upper GM, lower GM, BF, VL	None (EMG only)	10 reps per variation	MVIC
Bautista et al. [35]	Front squat vs. Overhead squat (+ plank + back extension)	65%, 80%, 95% of 3 RM	Below parallel	Barbell	Surface EMG	VL, BF, ES, MT, RA, EO, SA, AD	None (EMG only)	3 RM per intensity	MVIC
Knoll et al. [36]	SLS variation vs. Traditional split squat	Body weight	To max knee flexion	Force plate, wireless goniometer	Wireless surface EMG (Delsys Trigno)	VL, VM, RF, GM	Force plate (COP analysis)	1 set of 5 reps per variation	MVIC
van den Tillaar et al. [37]	Unilateral squat: free weight vs. Smith machine (foot forward vs. backward)	4 RM load	To ~90° knee flexion	Barbell, Smith machine, force plate, linear encoder	Surface EMG (Musclelab)	RF, VL, VM, BF, GM, GMed, Sol, MG, LG, ES, Add	Linear encoder + force plate kinematics	4 reps per condition	MVIC
Flores et al. [38]	Squat at 3 depths × 3 loads	Unloaded, 50%, 85% 1 RM	Above parallel, parallel, below parallel	Barbell, force platform, motion capture	Not used (kinetic focus)	Not assessed	3D kinematics + force plate; inverse dynamics (pEKFM)	3 reps per condition	N/A
Clark et al. [39]	Back squat at 85% 1 RM to volitional fatigue	85% 1 RM	Below parallel	Barbell, squat rack	Surface EMG	RA, EO, LSES, ULES, VL	Force plate	Set to failure	MVIC
Mackey et al. [40]	Bulgarian split squat (BSS) vs. Back squat (BS)	BSS: 35% 1 RM; BS: 70% 1 RM	Full depth	Barbell, bench, force plate, motion capture	Motion capture + force plate	Not assessed (kinetics focus)	3D inverse dynamics (ankle, knee, hip NJMI, NJW, NJM)	2 × 3 reps	N/A
Erman et al. [41]	Body weight squat to fatigue	Body weight	Self-selected (~90°)	4 load cell platforms	Force plates (4 load cells)	Not assessed	Load cell GRF + video ROM analysis	Repetitions to failure	N/A
Yoshiko and Watanabe [42]	Shallow squat vs. Deep squat (home-based 12-week training)	Body weight	SS: 60 cm target; DS: 40 cm target	Chair + cushion	Ultrasound; maximal isometric knee extension testing; physical function tests	Quadriceps femoris (VL, RF, VI via US)	Isometric torque at 30°, 70° and 110°; walking, sit-to-stand and leg-press tests	4 sets × 35 reps/day, 3 d/wk, 12 wks	N/A
Larsen et al. [43]	High bar narrow/wide vs. Low bar narrow/wide stance	3 RM	To parallel or below	Barbell (Rogue Ohio), force plate, Qualisys motion capture	Surface EMG (Musclelab, 1000 Hz)	ES-I, ES-L, GM, GMed, ST, BF, Add, RF, VL, VM, MG, Sol	3D kinematics (Qualisys) + force plate + linear encoder	3 RM attempts	MVIC
Sinclair et al. [44]	Narrow vs. Mid vs. Wide stance (Exp1: habitual; Exp2: manipulated)	70% 1 RM	Full depth	Barbell, 8-camera Qualisys, force platform	Musculoskeletal modeling (OpenSim)	Modeled forces: GM, BF, VL, VM, RF, Sol, MG	3 D kinematics + musculoskeletal model (OpenSim)	5 reps per condition	N/A
Kim et al. [45]	General squat (GS) vs. Wall squat (WS) vs. Spanish squat (SS)	Body weight	~90° knee flexion	Wall, elastic band (Spanish squat)	Surface EMG	RF, VL, VM, BF, ST, SM	None (EMG only)	5 reps per squat type	MVIC
Svensson et al. [46]	Free barbell squat vs. Smith machine (fixed path) squat	Body mass equivalent	90° knee flexion	Barbell, Smith machine	Textile EMG shorts (Myontec)	Quadriceps (group), Hamstrings (group), GM	None (EMG amplitude only)	5 reps per condition	Mean processed EMG amplitude in μV; no MVIC normalization
Lahti et al. [47]	Narrow vs. Wide stance back squat	70% and 85% 1 RM	Parallel depth	Barbell, 8-camera Vicon, 2 force plates	Not used (kinetics focus)	Not assessed	3 D inverse dynamics (hip and knee sagittal + frontal kinetics)	3 reps per stance	N/A

Abbreviations: MVIC, maximum voluntary isometric contraction; N/A, not applicable.

**Table 3:** Outcome-associated variables and overall inference.

Author(s)	Muscle Activation (EMG) Findings	Joint Kinematics Findings	Joint Kinetics/Moments	Ground Reaction Forces	Key Significant Findings (*p*-Values)	Effect Sizes	Potential Biomechanical Interpretation
Caterisano et al. [24]	GM relative contribution to total EMG increased with depth during the concentric phase. VL, VM, BF showed no significant difference across depths.	Greater hip and knee flexion in full squat compared to partial.	Not directly assessed.	Not assessed.	GM partial vs. full: *p* < 0.05	Not reported	Greater depth was associated with a greater relative gluteus maximus contribution.
Flanagan et al. [25]	VL, VM, MG, SOL: higher in SQ. BF, GM: higher in CSQ. TA similar.	SQ: greater knee and ankle ROM. CSQ: greater hip flexion.	SQ: higher knee extensor and ankle plantar flexor moments. CSQ: higher hip extensor moments.	Not directly reported.	Activity × phase interaction: *p* < 0.05 for multiple variables	Not reported.	CSQ shifted demand toward the hip; SQ toward the knee and ankle.
Gullett et al. [26]	No significant difference in any muscle EMG between front and back squat. Ascending phase EMG > descending (*p* < 0.05).	Not directly compared between squat types.	BS: significantly higher compressive forces and knee extensor moments (1.0 ± 0.4 vs. 0.7 ± 0.2 Nm/kg, *p* = 0.001). Shear forces similar.	Not separately reported.	Knee extensor moment: *p* = 0.001; Compressive force: *p* < 0.05	d not reported; mean diff = 0.3 Nm/kg	Front squat produced similar muscle activation with lower knee compressive loads and knee extensor moments.
Paoli et al. [27]	Only GM showed significant difference: higher at widest stance at 0% and 70% 1 RM. No difference for other 7 muscles across stance widths or loads.	Not assessed.	Not assessed.	Not assessed.	GM wide vs. narrow: *p* < 0.05 at 0% and 70% 1 RM	Not reported.	Wider stance was associated with greater gluteus maximus activation.
Escamilla et al. [28]	Not assessed (no EMG).	Wider-stance groups had greater hip flexion; shanks were more vertical. Trunk position did not differ significantly.	Wide-stance groups had greater hip and knee net muscle moments; narrow stance produced plantar-flexor and medium/wide stances dorsiflexor moments.	Not separately reported.	Significant hip/thigh position and 2D-versus-3D moment-arm differences; precise *p* values not extracted.	Not extracted.	Habitual stance groups differed biomechanically; this between-participant comparison does not establish effects of intentional stance modification.
Macrum et al. [29]	VL (*p* = 0.002, ES = 0.33) and VMO (*p* = 0.049, ES = 0.20) decreased with wedge. SOL increased (*p* = 0.03, ES = 0.64). LG unchanged.	Wedge: decreased peak knee flexion (*p* < 0.001, ES = 0.81), increased peak knee valgus (*p* = 0.02, ES = 0.21), increased MKD (*p* < 0.001, ES = 2.92).	Not assessed.	Not assessed.	Knee flexion: *p* < 0.001; MKD: *p* < 0.001	ES range: 0.20–2.92	Restricted ankle dorsiflexion was associated with greater knee valgus and medial knee displacement and lower quadriceps activation.
Bryanton et al. [30]	Not assessed by EMG; relative muscular effort was torque-derived.	Greater hip and knee flexion at deeper depths.	Relative knee extensor effort increased with depth but not load; ankle plantar-flexor effort increased with load but not depth; hip extensor effort increased with both.	Not separately reported.	Depth × load interactions for relative muscular effort: *p* < 0.05.	Effect sizes reported per variable	Depth and load altered joint-angle-specific muscular effort; this was not a muscle-specific EMG result.
Aspe and Swinton [31]	OHS: greater RA and EO (eccentric phase, *p* < 0.05, ~2–7% diff). BS: greater ES and all lower-body muscles (concentric, *p* < 0.05).	Not assessed.	BS produced significantly greater peak force (*p* < 0.05).	Peak force higher in BS.	RA, EO eccentric: *p* < 0.05; ES, GM, VL, BF concentric: *p* < 0.05	Magnitude ~2–7% MVIC for trunk	OHS did not substantially enhance trunk muscle activation vs. BS; BS elicited greater lower-body activation.
Andersen et al. [32]	GM, VL, RF: similar between bilateral squat and Bulgarian squat. GMed: higher in Bulgarian squat on unstable surface (*p* < 0.05). 6 RM strength lower on unstable surface.	Not assessed.	Not assessed.	Not assessed.	GMed unstable: *p* < 0.05; 6 RM stable vs. unstable: *p* < 0.01	Not reported.	Unstable surfaces increased stabilizer (GMed) demand but reduced the 6 RM load.
Yavuz et al. [33]	VM greater in FS ascending (*p* < 0.05, d = 0.62) and whole (*p* < 0.05, d = 0.41). ST greater in BS ascending (*p* < 0.05, d = −0.79).	BS: greater trunk lean (*p* < 0.05). No difference in knee kinematics.	Not directly reported.	Not assessed.	VM FS vs. BS ascending: *p* < 0.05; ST BS vs. FS ascending: *p* < 0.05	d: 0.41–0.79	FS elicited greater VM activity, and BS greater ST activity and trunk lean.
Contreras et al. [34]	No significant differences in upper GM, lower GM, BF, or VL EMG among parallel, full, and front squats (*p* > 0.05 for all).	Not assessed.	Not assessed.	Not assessed.	All comparisons: *p* > 0.05	Not significant	All three variations produced comparable muscle activation in trained females.
Bautista et al. [35]	AD greater in FS vs. OHS at 65% and 95% 3 RM (*p* < 0.05). MT greater in OHS at 65% and 80% 3 RM (*p* < 0.05). VL, BF: no difference.	Not assessed.	Not assessed.	Not assessed.	AD FS vs. OHS: *p* < 0.05; MT OHS vs. FS: *p* < 0.05	Not reported.	OHS elicited greater middle trapezius activity and FS greater anterior deltoid activity.
Knoll et al. [36]	VL, VM, RF: greater peak and mean EMG in traditional split squat (*p* < 0.05). GM: greater peak and mean in SLS variation (*p* < 0.01).	Not directly assessed.	Not assessed.	AP displacement of COP greater in SLS variation (*p* < 0.001, d = 4.309).	GM SLS vs. split: *p* = 0.002 (peak), *p* = 0.018 (mean); VL, VM, RF split > SLS: *p* < 0.05; COP: *p* < 0.001	d: 0.641–4.309	Split squat favored quadriceps activation and the SLS variation gluteus maximus activation, with greater COP displacement.
van den Tillaar et al. [37]	GM, VM lower in Smith machine foot-backwards (*p* < 0.05). GMed lower in Smith foot-forward vs. free-weight (*p* < 0.05). Sol higher Smith forward vs. backward and free forward (*p* < 0.05).	Peak barbell velocity increased across reps with free weight; fixed path in Smith.	Peak force higher in Smith machine (*p* < 0.05).	Peak force significantly higher in Smith machine condition.	GM, VM, MG Smith back < free: *p* < 0.05; Peak force Smith > free: *p* < 0.05	Not reported.	Free-weight unilateral squats elicited greater activation of several muscles, whereas the Smith machine allowed higher peak force.
Flores et al. [38]	Not assessed.	Greater knee flexion at deeper depths.	pEKFM increased with both depth and load (*p* ≤ 0.02). Slope of pEKFM greater from unloaded to 50% 1 RM vs. 50–85% 1 RM (*p* < 0.001).	Not separately reported.	pEKFM depth: *p* < 0.02; pEKFM load: *p* < 0.02; Slope difference: *p* < 0.001	Not reported.	Typical load reductions with deeper squats did not offset increases in knee extension moments.
Clark et al. [39]	VL EMG increased across fatiguing set (*p* < 0.05). Trunk muscles (RA, EO, LSES, ULES) increased with fatigue regardless of training status (*p* < 0.05). EG: lower normalized EMG despite higher absolute loads.	Not assessed.	Not assessed.	Not assessed.	VL increase with fatigue: *p* < 0.05; Trunk EMG increase: *p* < 0.05; EG vs. NG rep count: *p* < 0.05	Not reported.	Trunk and VL activation increased with fatigue; trained lifters showed lower normalized EMG at higher absolute loads.
Mackey et al. [40]	Not assessed (kinetics focus).	BSS: greater peak ankle displacement (*p* = 0.011); BS: greater peak knee displacement.	Hip NJMI, NJW, peak NJM significantly greater than knee and ankle in both squats (d = 5.50–9.40). BSS: knee NJMI and NJM less than ankle (d = 2.10–2.80). BS: knee NJMI > ankle.	Not separately reported.	Squat × joint interactions: *p* < 0.001 for all kinetic variables	d: 2.10–9.40 (very large effects)	BSS was more hip-dominant with lower relative knee demands than BS.
Erman et al. [41]	Not assessed.	Hip, knee, ankle ROM increased with fatigue progression (*p* < 0.05); ROM irregularities in late-fatigue phase.	Not assessed.	GRF distribution shifted with fatigue; fore-hind disparity decreased on support leg in late phase.	ROM increase with fatigue: *p* < 0.05; GRF shift: *p* < 0.05	Not reported.	Fatigue during bodyweight squats increased ROM and altered GRF distribution.
Yoshiko and Watanabe [42]	Not assessed (functional outcomes focus).	Not assessed.	Not assessed.	Not assessed.	Knee extension torque, quadriceps thickness and walking speed: no change (*p* > 0.05). Sit-to-stand and 1 RM leg press improved within both groups at selected times (*p* < 0.05).	Not reported.	Shallow and deep training showed selected within-group functional improvements; no overall superiority of deep squats or hypertrophy benefit was established.
Larsen et al. [43]	Wide stance: higher GM (poststicking), higher Add (sticking + poststicking) (*p* < 0.05). High bar: higher GM (sticking), higher VL (poststicking) (*p* < 0.05). Low bar: higher ES-I (all regions) (*p* < 0.05).	Low bar: greater trunk lean. Wide: greater lateral barbell displacement.	Low bar: higher peak force. Wide stance: higher peak force (*p* < 0.05).	Peak force higher in low bar and wide conditions.	GM, Add wide vs. narrow: *p* < 0.05; VL high bar vs. low bar: *p* < 0.05; ES-I low bar: *p* < 0.05	Not reported.	Bar placement and stance width altered muscle-specific activation (low bar: greater ES-I; high bar: greater VL).
Sinclair et al. [44]	Modeled forces, not EMG: wide stance increased posterior chain forces in both experiments; narrow stance increased quadriceps forces in experiment 2.	Wide stance: greater hip abduction. Narrow: greater knee flexion.	No significant differences in sagittal joint moments across stance widths.	Medial GRF impulse significantly greater in wide stance (both experiments, *p* < 0.05). Peak power greater in narrow stance (Exp2, *p* < 0.05).	Medial GRF: *p* < 0.05; Peak power narrow: *p* < 0.05	Reported per variable	Stance width altered modeled muscle forces; these estimates were not direct measures of activation or evidence of hypertrophy or clinical benefit.
Kim et al. [45]	SS: RF significantly greater than WS and GS (*p* < 0.001, η^2^ = 0.333). VL: SS greater than WS (*p* = 0.029, η^2^ = 0.109). No hamstring differences.	Not assessed.	Not assessed.	Not assessed.	RF SS vs. GS/WS: *p* < 0.001; VL SS vs. WS: *p* = 0.029	η^2^: 0.109–0.333	The Spanish squat elicited the highest quadriceps (especially RF) activation among GS, WS, and SS.
Svensson et al. [46]	Free path: greater whole-squat quadriceps activation (right MD 14 μV, *p* = 0.04; left MD 15 μV, *p* = 0.01); phase-specific hamstring differences; no GM difference.	Not directly compared.	Not assessed.	Not assessed.	Whole-squat quadriceps: *p* = 0.04/right and *p* = 0.01/left; phase-specific hamstrings: *p* = 0.03 and *p* = 0.02.	Quadriceps partial η^2^: 0.28/right and 0.39/left.	Free-path squats increased quadriceps and selected phase-specific hamstring activity at body-mass-equivalent loads; no rehabilitation benefit was tested.
Lahti et al. [47]	Not assessed (kinetics focus).	Narrow stance: greater knee flexion (*p* < 0.0001). Hip flexion did not differ at the corrected α = 0.01 threshold.	Wide stance: higher hip-to-knee extension moment ratio and knee adduction moment (*p* < 0.007).	Not separately reported.	Knee flexion: *p* < 0.0001; hip-to-knee moment ratio and knee adduction: *p* < 0.007; hip flexion: *p* = 0.049–0.109 (not significant at α = 0.01).	Knee flexion d = 2.56–2.86; kinetic differences d = 0.51–1.41.	Stance width changed sagittal demand ratios and frontal knee moments; increased hip flexion was not established.

**Table 4:** GRADE evidence profile for the main outcome domains.

Evidence Domain	Study Count (k)	Study Framework	Risk-of-Bias Concerns	Consistency Across Studies	Directness/Applicability	Precision	Publication/Reporting Concerns	Certainty Rating
Lower limb muscle activation across squat variations [24,25,26,27,29,31,32,33,34,35,36,37,39,43,45,46]	16	Predominantly cross-sectional or repeated measures laboratory studies, with crossover elements in some studies	Serious; objective sEMG was used, but several studies had small samples, incomplete confounder control, variable normalization procedures, and limited assessor blinding	Serious; findings differed by squat depth, stance width, load, bar position, unilateral status, fatigue state, and muscle group assessed	Serious; most samples were healthy, young, trained, and male, limiting transfer to older, female, injured, or rehabilitation populations	Serious; most studies had small sample sizes and no pooled estimate was available	Possible; small positive biomechanical studies may be more likely to be published	Very low
Joint kinematics and range-of-motion outcomes [24,25,28,29,30,33,37,38,40,41,43,44,47]	13	Mostly cross-sectional or repeated measures biomechanical studies using video analysis, motion capture, or related movement-analysis systems	Serious; measurement tools were generally objective, but study protocols, depth definitions, and technical control varied	Serious; kinematic responses were condition-specific and varied across depth, stance, bar placement, fatigue, and unilateral/bilateral tasks	Serious; most data came from controlled laboratory squats rather than clinical rehabilitation or sport-specific longitudinal settings	Serious; small samples and heterogeneous reporting prevented precise quantitative certainty	Possible; selective reporting of significant joint-angle changes could not be excluded	Very low
Joint kinetics, joint moments, and internal/external loading variables [25,26,28,30,38,40,43,44,47]	9	Cross-sectional or repeated measures studies using force plates, inverse dynamics, or musculoskeletal modeling	Serious; objective kinetic methods were used, but inverse-dynamics/modeling assumptions and protocol heterogeneity reduced confidence	Serious; kinetic findings differed by load, stance, depth, bar placement, and unilateral status	Serious; evidence mainly represented acute mechanical loading in healthy or trained adults, not long-term clinical outcomes	Serious; small samples, different units, and non-uniform reporting limited estimate precision	Possible; publication bias could not be evaluated formally	Very low
Ground reaction force and force-distribution outcomes [31,37,41,43,44]	5	Cross-sectional or repeated measures force-plate or load-cell studies	Serious; measurement was objective, but protocols differed markedly and some studies had limited reporting of confounding control	Serious; force responses were dependent on equipment, stance, fatigue, and free-weight versus machine-based conditions	Serious; GRF outcomes were surrogate biomechanical variables and were not directly linked to injury or functional recovery	Very serious; few studies contributed data and sample sizes were small	Possible; evidence base was narrow and vulnerable to selective reporting	Very low
Functional, fatigue, balance, strength and muscle-thickness outcomes [32,36,39,41,42]	5	One randomized trial plus cross-sectional or fatigue/balance-oriented studies	Serious; the randomized trial was limited by small sample size and some outcome/reporting concerns, while non-randomized studies had design-related limitations	Serious; acute load capacity, COP and fatigue variables differed from longitudinal strength/function outcomes. The trial did not establish depth superiority or increased muscle thickness.	Serious; functional outcomes were sparse and mostly surrogate, with limited representation of patients or sport-specific follow-up	Very serious; evidence was based on few studies and no pooled functional estimate was possible	Possible; small-study effects and selective reporting could not be excluded	Very low

**Table 5:** Summary of findings.

Assessed Outcome	Narrative Findings (No Absolute Effect Estimate)	Comparative/Relative Effect	Participants and Studies	Evidence Certainty (GRADE)
Muscle activation response to squat variation	Depth-related GM activation varied; wider stance increased GM activation in some protocols. Front/back activity was often similar; free/fixed paths showed muscle- and phase-specific differences.	Not pooled; qualitative direction favored variation-specific recruitment rather than universal superiority of one squat type	Study-level *n* = 268 across 16 studies [24,25,26,27,29,31,32,33,34,35,36,37,39,43,45,46]	Very low
Joint kinematic changes	Greater squat depth was associated with greater hip and knee flexion; wider stance tended to increase hip abduction/flexion while reducing knee flexion and ankle dorsiflexion in some protocols; fatigue increased joint excursion	Not pooled; direction of effect was condition-dependent and varied by squat type, stance, and fatigue status	Study/experiment-level *n* = 312 across 13 reports [24,25,28,29,30,33,37,38,40,41,43,44,47]	Very low
Joint kinetic and loading changes	Greater depth/load increased selected knee extensor demands; stance changed hip-to-knee moment ratios, without consistently lowering knee loading. One front/back comparison found lower knee compression in front squats.	Not pooled; evidence suggested redistribution of hip-knee loading rather than a uniform increase or decrease in total mechanical demand	Study/experiment-level *n* = 241 across 9 reports [25,26,28,30,38,40,43,44,47]	Very low
Ground reaction force and force-distribution changes	Force output and impulse distribution changed according to free-weight versus machine condition, stance width, bar placement, and fatigue progression	Not pooled; available evidence suggested measurable but protocol-specific changes in force production and distribution	Study/experiment-level *n* = 153 across 5 reports [31,37,41,43,44]	Very low
Functional, fatigue, balance, strength and muscle-thickness outcomes	Acute fatigue, COP and 6 RM findings were indirect. Both trial groups improved selected functional tests from baseline; torque/thickness/walking speed did not significantly change, and depth superiority was not established.	Not pooled; functional evidence remained sparse and was not sufficient to establish durable training or rehabilitation superiority	Study-level *n* = 108 across 5 studies [32,36,39,41,42]	Very low

Abbreviations: COP, center of pressure; EMG, electromyography; GRADE, Grading of Recommendations Assessment, Development, and Evaluation; GRF, ground reaction force; ROM, range of motion; sEMG, surface electromyography. Participant totals in Table 5 sum the sample sizes listed in Table 1 for contributing reports/experiments; they are not pooled effect estimates or verified counts of unique individuals. Sinclair et al. [44] contributed experiments of *n* = 70 and *n* = 20.

## Data Availability

No new data were created or analyzed in this study. Data sharing is not applicable to this article.

## Supplementary Materials

The following supporting information can be downloaded at https://www.mdpi.com/article/10.3390/healthcare14213561/s1, Table S1: Database specific search strategies; Table S2: PRISMA checklist [56].
