# Supplementary material for: Effects of Squat Exercise Variations on Lower Limb Biomechanics and Functional Outcomes: Implications for Physiotherapy and Rehabilitation: A Systematic Review
Source: Healthcare (Basel). 2026 Oct 22;14(21):3561. doi: 10.3390/healthcare14213561 (PMC13763054; doi:10.3390/healthcare14213561)
Supplement: Supplementary file 1 [file healthcare-14-03561-s001.zip › Table S1.pdf]

Table S1: Database specific search strategies

Database and platform	Search string	Search date
**PubMed/MEDLINE**<br>PubMed	(squat*[tiab]) AND (electromyograph*[tiab] OR EMG[tiab] OR myoelectric*[tiab] OR "muscle activation"[tiab] OR "muscular activation"[tiab] **OR** biomechanic*[tiab] OR kinematic*[tiab] OR kinetic*[tiab] OR "joint angle*"[tiab] OR "range of motion"[tiab] OR ROM[tiab] OR moment*[tiab] OR torque*[tiab] OR force*[tiab] OR GRF[tiab] OR load*[tiab] OR pressure*[tiab] OR power[tiab] OR "center of pressure"[tiab] OR "centre of pressure"[tiab] OR COP[tiab] OR function*[tiab] OR strength[tiab] OR balance[tiab] OR jump*[tiab] OR performance[tiab] OR fatigue[tiab] OR pain[tiab] OR hypertroph*[tiab] OR "muscle thickness"[tiab])	From inception till 1st April, 2026
**Embase**<br>Embase.com	(squat*:ti,ab,kw) AND (electromyograph*:ti,ab,kw OR EMG:ti,ab,kw OR myoelectric*:ti,ab,kw OR 'muscle activation':ti,ab,kw OR 'muscular activation':ti,ab,kw **OR** biomechanic*:ti,ab,kw OR kinematic*:ti,ab,kw OR kinetic*:ti,ab,kw OR 'joint angle*':ti,ab,kw OR 'range of motion':ti,ab,kw OR ROM:ti,ab,kw OR moment*:ti,ab,kw OR torque*:ti,ab,kw OR force*:ti,ab,kw OR GRF:ti,ab,kw OR load*:ti,ab,kw OR pressure*:ti,ab,kw OR power:ti,ab,kw OR 'center of pressure':ti,ab,kw OR 'centre of pressure':ti,ab,kw OR COP:ti,ab,kw OR function*:ti,ab,kw OR strength:ti,ab,kw OR balance:ti,ab,kw OR jump*:ti,ab,kw OR performance:ti,ab,kw OR fatigue:ti,ab,kw OR pain:ti,ab,kw OR hypertroph*:ti,ab,kw OR 'muscle thickness':ti,ab,kw)	
**Scopus**<br>Advanced search	TITLE-ABS-KEY(squat*) AND TITLE-ABS-KEY(electromyograph* OR EMG OR myoelectric* OR "muscle activation" OR "muscular activation" **OR** biomechanic* OR kinematic* OR kinetic* OR "joint angle*" OR "range of motion" OR ROM OR moment* OR torque* OR force* OR GRF OR load* OR pressure* OR power OR "center of pressure" OR "centre of pressure" OR COP OR function* OR strength OR balance OR jump* OR performance OR fatigue OR pain OR hypertroph* OR "muscle thickness")	
**Web of Science Core Collection**<br>Advanced search	TS=(squat*) AND TS=(electromyograph* OR EMG OR myoelectric* OR "muscle activation" OR "muscular activation" **OR** biomechanic* OR kinematic* OR kinetic* OR "joint angle*" OR "range of motion" OR ROM OR moment* OR torque* OR force* OR GRF OR load* OR pressure* OR power OR "center of pressure" OR "centre of pressure" OR COP OR function* OR strength OR balance OR jump* OR performance OR fatigue OR pain OR hypertroph* OR "muscle thickness")	
**CINAHL Ultimate**<br>EBSCOhost	(TI squat* OR AB squat*) AND (TI (electromyograph* OR EMG OR myoelectric* OR "muscle activation" OR "muscular activation" **OR** biomechanic* OR kinematic* OR kinetic* OR "joint angle*" OR "range of motion" OR ROM OR moment* OR torque* OR force* OR GRF OR load* OR pressure* OR power OR "center of pressure" OR "centre of pressure" OR COP OR function* OR strength OR balance OR jump* OR performance OR fatigue OR pain OR hypertroph* OR "muscle thickness") OR AB (electromyograph* OR EMG OR myoelectric* OR "muscle activation" OR "muscular activation" **OR** biomechanic* OR kinematic* OR kinetic* OR "joint angle*" OR "range of motion" OR ROM OR moment* OR torque* OR force* OR GRF OR load* OR pressure* OR power OR "center of pressure" OR "centre of pressure" OR COP OR function* OR strength OR balance OR jump* OR performance OR fatigue OR pain OR hypertroph* OR "muscle thickness"))	

**SPORTDiscus**<br>EBSCOhost	(TI squat* OR AB squat*) AND (TI (electromyograph* OR EMG OR myoelectric* OR "muscle activation" OR "muscular activation" **OR** biomechanic* OR kinematic* OR kinetic* OR "joint angle*" OR "range of motion" OR ROM OR moment* OR torque* OR force* OR GRF OR load* OR pressure* OR power OR "center of pressure" OR "centre of pressure" OR COP OR function* OR strength OR balance OR jump* OR performance OR fatigue OR pain OR hypertroph* OR "muscle thickness") OR AB (electromyograph* OR EMG OR myoelectric* OR "muscle activation" OR "muscular activation" **OR** biomechanic* OR kinematic* OR kinetic* OR "joint angle*" OR "range of motion" OR ROM OR moment* OR torque* OR force* OR GRF OR load* OR pressure* OR power OR "center of pressure" OR "centre of pressure" OR COP OR function* OR strength OR balance OR jump* OR performance OR fatigue OR pain OR hypertroph* OR "muscle thickness"))	

(Note: Limits: Search from inception through the execution date. Apply English-language, human-study and original peer-reviewed-report eligibility during screening. Do not restrict age, clinical population, rehabilitation, study design or full-text availability in the database search)
